# Efficacy of Calcium Hydroxide/Saline versus Calcium Hydroxide/*Artemisia persica* Essential Oil as Intracanal Medicament to Improve Radiographic Visualization of Periapical Lesions in Necrotic Teeth: A Randomized Clinical Trial

**DOI:** 10.1155/2023/6364881

**Published:** 2023-02-16

**Authors:** Ameneh Shareghi, Elmira Ahmadpoor, Zahra Lorigooini, Farnoosh Ahmadi, Saba Tohidkhah

**Affiliations:** ^1^Department of Endodontics, Faculty of Dentistry, Shahrekord University of Medical Science, Shahrekord, Iran; ^2^Medical Plants Research Center, Basic Health Science Institute, Shahrekord University of Medical Science, Shahrekord, Iran; ^3^Shahrekord University of Medical Sciences, Shahrekord, Iran; ^4^Minnesota Dental Research Center for Biomaterials and Biomechanics, School of Dentistry, University of Minnesota, Minneapolis, MN, USA; ^5^Tehran University of Medical Sciences, Tehran, Iran

## Abstract

**Objectives:**

This study aimed to compare the efficacy of calcium hydroxide (CH)/saline and CH/*Artemisia persica* (*A. persica*) essential oil as an intracanal medicament for radiographic resolution of periapical (PA) lesions in necrotic teeth.

**Materials and Methods:**

This randomized clinical trial was conducted on 22 patients with necrotic teeth and PA lesions presenting to two private endodontic offices. The patients were randomly divided into two groups (*n* = 11) to receive CH/saline (control group) and CH/*A. persica* essential oil (10%) (intervention group) as intracanal medicaments between treatment sessions. The size of PA radiolucency was measured on parallel PA radiographs taken before treatment and also at 1 and 3 months after completion of treatment. The mean time of healing of PA lesions was also compared between the two groups. Data were analyzed by the independent *t*-test, chi-square test, and Fisher's exact test (alpha = 0.05).

**Results:**

No significant difference was found between the two groups regarding the changes in the size of PA lesions, relative healing percentage, and speed of healing, neither at 1 nor at 3 months postoperatively (*P* > 0.05). Regarding the presence/absence of clinical symptoms in the second treatment session, the intervention group showed greater resolution of symptoms, although the difference did not reach statistical significance (*P* > 0.05).

**Conclusion:**

According to the present results, it appears that the addition of *A. persica* essential oil to CH for application as intracanal medicament does not add any particular advantage.

## 1. Introduction

The main purpose of endodontic treatment is to eliminate the bacteria and necrotic products from the root canal system and prevent the leakage of microorganisms into the periapical tissue. However, despite optimal cleaning and shaping, some resistant bacterial species may remain in the root canal system and cause refractory infections [1, 2].

Apical periodontitis refers to inflammation of the periodontal ligament in the periapical (PA) region. PA lesions are caused by the local response of PA tissues to pulpal infection or necrosis and are characterized by inflammation and possible bone resorption at the root apex. PA lesions often heal following the elimination of the cause. The majority of PA lesions develop following pulpal necrosis in nonvital teeth. In the case of vital teeth, apical periodontitis may occur due to mild occlusal trauma, bruxism, or pressure applied in orthodontic treatment [3, 4]. Apical periodontitis commonly occurs in endodontically treated teeth, and its prevalence increases with age [5]. Suboptimal endodontic treatment is one of the most important causes of apical periodontitis [6, 7]. Microorganisms, their endotoxins and exotoxins, enzymes, and the host immune response are involved in the development of PA lesions [5]. Activation of osteoclasts leads to bone resorption in the periapical area when bacteria create a favorable environment.

Resistant bacteria such as *Enterococcus faecalis* (*E. faecalis*) can prolong the healing course of PA lesions [8]. *E. faecalis* is commonly isolated from teeth with failed endodontic treatment [9]. This microorganism can tolerate difficult environmental conditions such as highly alkaline pH, dryness, and high concentrations of salt. It can also form a biofilm in root canals, which confers resistance against antimicrobial agents [10]. *E. faecalis* is a Gram-positive anaerobic coccus that is found in 30–90% of endodontically treated root canals [11, 12]. Both *E. faecalis* and *Candida albicans* (*C. albicans*) have specific properties that help them survive in endodontically treated root canals, such as resistance to intracanal medicaments, biofilm formation, invading the dentinal tubules, and surviving long periods of nutrient deprivation [13, 14]. The emergence of drug-resistant bacteria that can cause serious infections highlights the need to re-evaluate the currently used antimicrobial agents and search for novel antimicrobials with higher efficacy and fewer side effects.

Calcium hydroxide (CH) is the most commonly used intracanal medicament between the treatment sessions in teeth requiring multisession endodontic treatment [15]. It is delivered into the root canals in the form of a paste, in combination with liquids such as saline or chlorhexidine. The mechanism of antimicrobial action of CH is not clearly understood. However, one possible mechanism is through the release of hydroxyl ions that raise the pH [16]. Most pathogenic bacteria cannot tolerate alkaline conditions [17]. Nonetheless, information regarding the antibacterial activity of CH is controversial. A previous study showed that the antibacterial activity of CH is inhibited by dentin [18]. Another clinical study showed that the number of Gram-positive microorganisms such as *E. faecalis* and *C. albicans* did not decrease after intracanal application of CH [19]. Also, another study showed insignificant efficacy of CH one week after its application [15]. Moreover, the use of CH has some limitations. It cannot completely eliminate all microorganisms from the root canal system and requires a long time to exert its antimicrobial effects [13]. Also, it is potentially toxic due to its high pH and can damage soft tissue by denaturing proteins, leading to chronic inflammation and cellular necrosis in the PA region [20, 21]. Furthermore, the buffering capacity of dentin decreases the release of hydroxyl ions and impedes the antimicrobial efficacy of CH [21]. Thus, attempts are ongoing to find novel materials with optimal antimicrobial efficacy for the complete removal of microorganisms from the root canal system.

The essential oils of medicinal herbs with antimicrobial activity may be a good candidate for this purpose. *Artemisia persica* (*A. persica*) is a valuable medicinal herb [22] with antioxidant [23], anticancer [24], antibacterial [25], antifungal [26], and antimalarial [26] effects. Cineol, camphor, and sesquiterpene are the three most abundant constituents of *A. persica* essential oil [27], which have antibacterial activity against *Escherichia coli*, *Staphylococcus aureus*, *Staphylococcus epidermidis*, and *C. albicans* [27, 28]. A previous study showed that *A. persica* mouthwash had optimal healing effects on patients with denture stomatitis [29]. Considering all the above, this study aimed to compare the efficacy of CH/saline and CH/*A. persica* essential oil as an intracanal medicament for radiographic resolution of PA lesions in necrotic teeth.

## 2. Materials and Methods

This study was conducted at Shahrekord University of Medical Sciences between January 2021 and October 2021. The study was approved by the ethics committee of the university (IR.SKUMS.REC.1399.186).

### 2.1. Trial Design

A randomized single-blind clinical trial was conducted in which the intervention group received CH/*A. persica* essential oil as intracanal medicament, while the control group received CH/saline as intracanal medicament between endodontic treatment sessions. The results were reported in accordance with the guidelines of the Consolidated Standards of Reporting Trials [30].

### 2.2. Participants, Eligibility Criteria, and Settings

The inclusion criteria were (i) maxillary or mandibular teeth with necrotic pulp and a PA lesion as diagnosed by clinical examination and radiography, (ii) completely formed apices, (iii) straight roots without severe curvature, and (iv) the presence of PA lesions with a minimum diameter of 2 mm.

The exclusion criteria were (i) teeth with cracks or fractures, root caries, or shape and size anomalies, (ii) history of previous endodontic treatment, (iii) severe mobility, and (iv) advanced periodontitis.

The sample consisted of 22 patients with necrotic teeth and PA radiolucency presenting to two endodontic offices in Shahrekord, Iran.

### 2.3. Interventions

The *A. persica* medicinal herb was collected from the mountains of Chaharmahal-Bakhtiari Province of Iran, and after identification in the herbarium of the Medicinal Plant Research Center of Shahrekord University of Medical Sciences, its essential oil was obtained by the Clevenger apparatus through water distillation. Dehydration was performed with sodium sulfate, and the essential oil was stored in a dark glass container away from sunlight and refrigerated at 4°C. Next, 10% *A. persica* essential oil was prepared in saline and hand-mixed until it mixed completely and then vortex-mixed for 60 seconds. A total of 22 patients were selected among those presenting to two private endodontic offices by convenience sampling. In other words, we selected those participants who were present at the time of the study. This is called convenience sampling because the people who took part were easy to find and willing to take part. The patients had necrotic teeth with PA radiolucency. Clinical assessment and pulp vitality tests confirmed pulp necrosis, and radiographic examination confirmed the presence of PA radiolucency. The size of the lesion was measured on the preoperative PA radiograph with no magnification and recorded. The lesion size was measured by an endodontist with more than a 5-year experience of using the digital caliper on the 164 negatoscope and recorded. The local anesthetic injection was administered, and after rubber-dam isolation, an access cavity was prepared. A K-file (Mani, Japan) was used to measure the working length, which was confirmed radiographically and also by an apex locator. The root canals were instrumented with hand files up to #30–40 for molar teeth and #40–50 for anterior and premolar teeth. Coronal shaping was performed using the crown-down technique. After using each file, the root canals were rinsed with 0.5 cc of 5.25% sodium hypochlorite. After completion of instrumentation, a final rinse with saline was performed with the syringe tip located at 2 mm from the working length. The root canals were then cleaned with paper points. The dental clinician was unaware of the type of medicament delivered into the canal until the completion of cleaning and shaping. After completion of cleaning and shaping, based on the code allocated to each patient, CH mixed with saline or 10% *A. persica* extract was prepared by the dental assistant and handed to the dental clinician to deliver into the canal by a Lentulo and paper points. To prepare the pastes, the required amount of CH was placed on a glass slab and mixed with either saline alone or saline mixed with 10% *A. persica* essential oil until a creamy consistency was achieved. After delivery of the paste into the canal, the access cavities were sealed by placing a dry cotton pellet and using temporary restorative material (Cavit). The patients were recalled after one week for completion of treatment. The intracanal medicament was removed with a file and irrigation with sodium hypochlorite. The root canals were dried with paper points and filled with gutta-percha and AH26 sealer. The temporary restoration was performed with Zonalin, and the patients were referred for the permanent restoration. The follow-up sessions were scheduled for 1 and 3 months after endodontic treatment. Clinical and radiographic examinations (parallel PA radiography) were performed at the follow-up sessions, and the size of PA radiolucency was measured on the follow-up radiographs and compared with its preoperative size. Measurements and interpretation of radiographs were all performed by the same endodontist. The intraexaminer reliability was examined via the intraclass correlation coefficient (ICC) and Kappa for quantitative and qualitative variables, respectively. The ICC values were >0.90, and all the Kappa scores were higher than 0.80. The presence/absence of clinical symptoms, such as redness and swelling at the site of the respective teeth, was also evaluated at the second treatment session.

### 2.4. Outcomes (Primary and Secondary)

The main objective of this study was to compare the efficacy of CH/saline and CH/*A. persica* essential oil as an intracanal medicament for reduction of the size of PA lesions. The speed of healing of PA lesion was evaluated by comparing the clinical symptoms of patients in the first and second treatment sessions. Redness and swelling were evaluated with inspection and palpation of the soft tissue, and pain was assessed with a percussion test. The radiographic and clinical assessments were made according to the following criteria modified from evaluation procedures used by Weiger et al. [31] and Çalışkan [32]. Complete healing (success): no clinical signs and symptoms and complete disappearance of preexisting radiolucency on radiography. Incomplete healing: no clinical signs and symptoms and, radiographically, a reduction in the size of the periapical radiolucency. No healing (failure): clinical signs and/or symptoms and radiographical expansion or no change in size of the preexisting lesion.

### 2.5. Sample Size Calculation

The sample size was calculated to be a minimum of 10 in each group according to a study by Kala and Sindhuri [33], assuming the mean change in the size of PA radiolucency of necrotic teeth to be 2.58 mm with a standard deviation of 1.6 mm in group 1 and 1.7 mm in group 2, alpha = 0.05, and beta = 0.10.

### 2.6. Randomization

A total of 20 patients were selected by convenience sampling and were randomly divided into two groups using the using simple random allocation. A random sequence was generated online (https://www.random.org) using a simple randomization technique by an independent person. The allocation was concealed by placing the sequence in a sealed envelope, which was then given to the dental assistant.

### 2.7. Blinding

The study had a single-blind design, and the patients were unaware of the type of medicament applied to their root canals.

### 2.8. Statistical Analysis

The independent *t*-test, chi-square test, and Fisher's exact test were applied to analyze the data by using SPSS version 16 at 0.05 level of significance. The independent *t*-test was used to identify statistical differences between the mean values of the groups. The chi-square test was used to determine the distribution of gender in two groups. Fisher's exact test was to analyze the frequency of clinical symptoms in two groups.

## 3. Results

**Figure 1:**
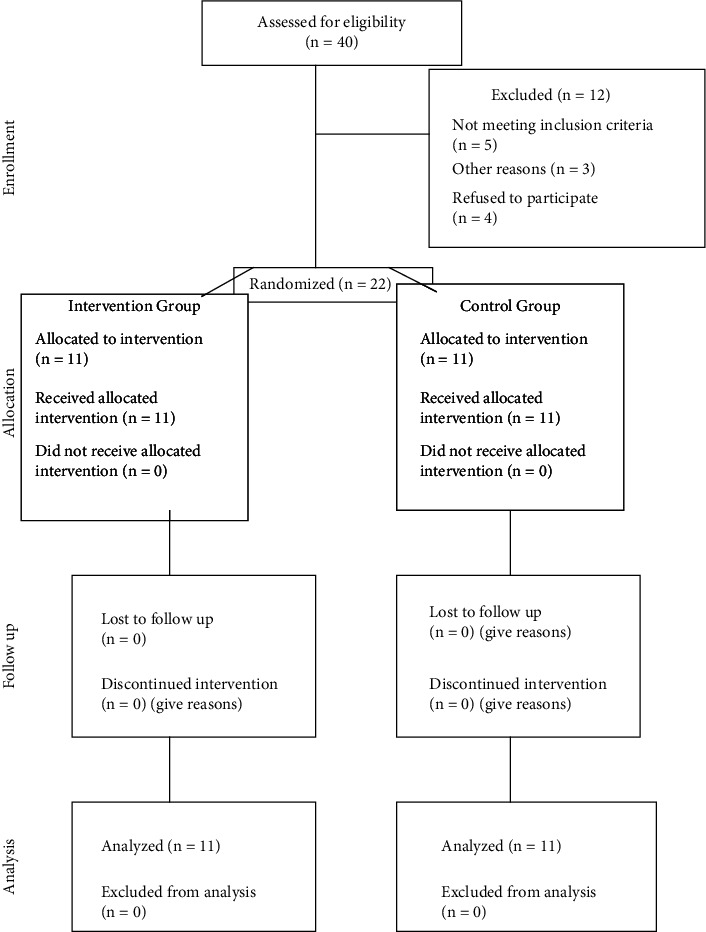
CONSORT diagram showing the flow of participants through each stage of a randomized trial.

The sample consisted of 22 patients, including 7 females and 5 males in the intervention group and 8 females and 4 males in the control group (*P* = 0.99).  Figure 1 presents the flow diagram of the study.

### 3.1. Harms

No patients were harmed during the study.

### 3.2. Group Analysis

#### 3.2.1. Mean Size of PA Lesions

Table 1 presents the mean size of PA lesions in millimeters at baseline and at 1 and 3 months after endodontic treatment in the two groups. The two groups had no significant difference regarding the mean size of PA lesions at any time point (*P* > 0.05).

#### 3.2.2. The Relative Percentage of Healing Based on the Difference in Radiographic Lesion Size

Table 2 presents the relative percentage of recovery at 1 and 3 months, compared with baseline, in the two groups. The two groups had no significant differences in this respect either (*P* > 0.05).

#### 3.2.3. Presence/Absence of Clinical Symptoms

Table 3 presents the frequency of clinical symptoms in the two groups during the second treatment session. As shown, the difference in this regard was not significant between the two groups (*P* = 0.64).

#### 3.2.4. Speed of Healing (the Change of the Lesion Size (mm) after 1 month and 3 months (mm/month))

Table 4 presents the mean speed of healing in the two groups at 1 and 3 months. As shown, the difference between the two groups was not significant in this parameter at any follow-up time (*P* > 0.05).

## 4. Discussion

This study compared the efficacy of CH/saline and CH/*A. persica* essential oil as intracanal medicament to improve radiographic visualization of PA lesions in necrotic teeth. The results showed no significant difference between the two groups regarding changes in the size of PA lesions, the relative percentage of healing, or the speed of healing, neither at 1 nor at 3 months postoperatively. Regarding the presence/absence of clinical symptoms in the second treatment session, the CH/*A. persica* essential oil group showed higher resolution of symptoms, although the difference did not reach statistical significance. The shorter follow-up period in the current study may have contributed to these results. It might take longer than 1–3 months to notice changes in lesion size on radiographs. According to Weiger et al. [31], the likelihood of periapical repair in its entirety with calcium hydroxide as an intracanal medication, including the 95% confidence interval, is 0.33 after one year of follow-up, 0.62 after two years, and 0.93 after five years. On the other hand, the size of the periapical lesion was demonstrated to be a risk factor in the same study. Accordingly, a larger periapical lesion had a lower likelihood of disappearing in a certain amount of time than a smaller lesion. However, it must be remembered that a radiograph's measurement of a lesion's diameter only approximates its actual dimensions.

Kaur and Benipal [34], in a clinical trial, compared the antimicrobial efficacy of octenidine dihydrochloride and *Artemisia annua* (*A. annua*) as root canal irrigants in endodontic treatment of maxillary central incisors and showed a reduction in microbial count in root canals irrigated with octenidine dihydrochloride. They found that octenidine dihydrochloride was more effective than *A. annua*, both alone and in combination with saline. Teoh et al. [35] showed that changing the composition of CH by adding a solvent changed the release of hydroxyl ions. This effect can be because different materials have different capacities for creating an alkaline environment in dentinal tubules and exerting antibacterial activity. They showed that, in contrast to using water as a solvent, a paste made using a water-free solvent produced a more alkaline pH in the dentin of extracted teeth. In our study, we found that adding CH as a solvent to *A. persica* essential oil did not significantly alter the outcomes. Although we have not evaluated the change in pH values in our study, it is possible that the lack of a significant difference between groups was caused by the fact that there was not much of a change in pH after adding *A. persica*.

Since *A. persica* as an intracanal medication had not previously been evaluated in either in vitro or in vivo studies, we attempted to compare our findings with those from studies that had used particularly similar materials. For instance, in Thøfner et al.' [36] study, five different *Artemisia annua*-derived materials were screened for their in vitro and in vivo activities against six clonal cultures of *Histomonas meleagridis*. In vitro, they discovered that *A. persica* inhibited the growth of *E. faecalis* and *C. albicans*, but in the clinical setting and root canal space, it had less of an impact. In addition, although *A. persica* has been shown to have significant antimicrobial and anti-inflammatory properties in vitro against *Pseudomonas aeruginosa, Enterococcus, Staphylococcus aureus*, and *Candida albicans* [25, 28, 37], we were unable to detect the same significance in our in vivo study. This finding might be explained by the fact that the intracanal medication can easily be washed out of the canal in a mouth environment, lowering the effective concentration of the substance inside the root canal. On the other hand, both *E. faecalis* and *C. albicans* can survive in endodontically treated teeth even after mechanical cleaning of the teeth, which makes the situation very different from an in vitro study [13, 14]. Therefore, we suggest that future research studies consider *A. persica* at higher concentrations. About the effect of different concentrations of Artemisia, Najafi et al. [38] evaluated the antimicrobial properties of *Artemisia deserti* and observed that different concentrations of the extract had different effects on *Malassezia furfur* and created growth inhibition zones with different diameters. With an increase in the concentration of the extract, the diameter of growth inhibition zones increased.

Future research is necessary to evaluate in vivo the antimicrobial effects of *A. persica* alone (without CH) and at various concentrations. Future research with a larger sample size is also necessary to get more accurate results.

## 5. Conclusion

According to the present results, it appears that the addition of *A. persica* essential oil to CH for application as intracanal medicament does not add any particular advantage.

## Figures and Tables

**Table 1:** Mean size of PA lesions at baseline and at 1 and 3 months after endodontic treatment in the two groups.

Group	Baseline		1 month		3 months	
	Median	Interquartile range	Median	Interquartile range	Median	Interquartile range
Control	7	5–7	4	3–5	2	0–3
Intervention	6.5	4–8	4	2–6	1	0–5
*P* value	0.89		0.82		0.92	

**Table 2:** Relative percentage of healing at 1 and 3 months, compared with baseline in the two groups.

Group	Baseline-1 month difference		Baseline-3 months difference	
	Median	Interquartile range	Median	Interquartile range
Control	33.3	25–42.9	50	66.7–100
Intervention	38.5	25–57.1	80	50–100
*P* value	0.53		0.82	

**Table 3:** The frequency of clinical symptoms in the two groups at the second treatment session.

Variable		Control	Intervention	*P* value
Asymptomatic	Number	7	9	0.64
	Percentage	63.6%	81.8%	
Redness/swelling	Number	4	2	
	Percentage	36.4%	18.2%	
Total	Number	11	11	
	Percentage	100%	100%	

**Table 4:** Mean speed of healing in the two groups at 1 and 3 months (*n* = 11 in each group).

Mean speed	Group	Mean	Std. deviation	*P* value
1 month	Control	1.86	0.61	0.44
	Intervention	2.1	0.94	

3 months	Control	1.54	0.49	0.83
	Intervention	1.49	0.50	

## Data Availability

The data used to support the findings of this study are available from the corresponding author upon request.
